# ^1^H, ^13^C, and ^15^N Backbone assignments of the human brain and acute leukemia cytoplasmic (BAALC) protein

**DOI:** 10.1007/s12104-020-09938-7

**Published:** 2020-04-02

**Authors:** Andras Lang, Amit Kumar, Jan Jirschitzka, Frank Bordusa, Oliver Ohlenschläger, Christoph Wiedemann

**Affiliations:** 1 Leibniz Institute on Aging – Fritz Lipmann Institute, Beutenbergstr. 11, 07745 Jena, Germany; 2 Department of Chemistry, Institute of Biochemistry, University of Cologne, Zülpicher Str. 47, 50674 Cologne, Germany; 3 Institute of Biochemistry and Biotechnology, Charles Tanford Protein Center, Martin-Luther-University Halle-Wittenberg, Kurt-Mothes-Str. 3a, 06120 Halle, Germany

**Keywords:** Resonance assignments, Heteronuclear NMR, Brain and acute leukemia cytoplasmic protein (BAALC), Intrinsically disordered protein (IDP), Neuroectodermal and hematopoietic cell function

## Abstract

The brain and acute leukemia cytoplasmic (BAALC; UniProt entry Q8WXS3) is a 180-residue-long human protein having six known isoforms. BAALC is expressed in either hematopoietic or neuroectodermal cells and its specific function is still to be revealed. However, as a presumably membrane-anchored protein at the cytoplasmic side it is speculated that BAALC exerts its function at the postsynaptic densities of certain neurons and might play a role in developing cytogenetically normal acute myeloid leukemia (CN-AML) when it is highly overexpressed by myeloid or lymphoid progenitor cells. In order to better understand the physiological role of BAALC and to provide the basis for a further molecular characterization of BAALC, we report here the ^1^H, ^13^C, and ^15^N resonance assignments for the backbone nuclei of its longest hematopoietic isoform (isoform 1). In addition, we present a ^1^H^N^ and ^15^N^H^ chemical shift comparison of BAALC with its shortest, neuroectodermal isoform (isoform 6) which shows only minor changes in the ^1^H and ^15^N chemical shifts.

## Biological context

**Fig. 1:**
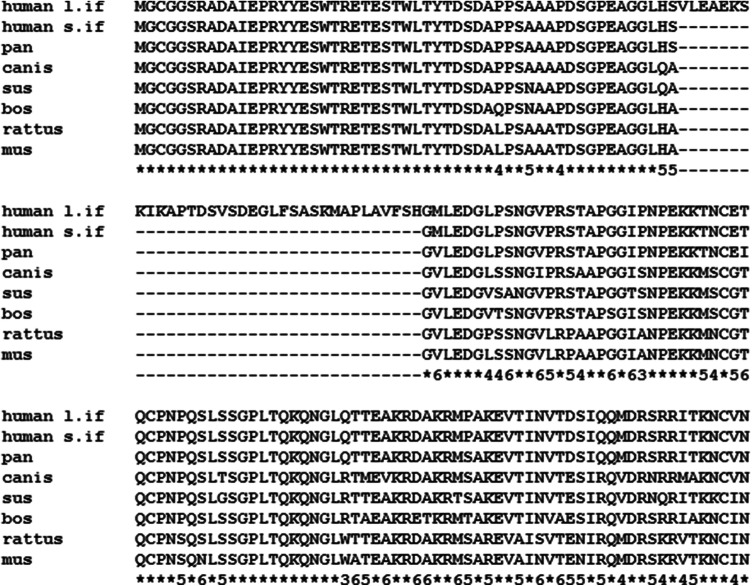
Sequence alignment of BAALC from selected species. First line is the longest human isoform 1 (l.if.), while all other lines are isoform 2 (s.if.). The last line that indicates the conservation: Asterisks = fully conserved; numbers are the occasions of the most frequent residue out of seven isoform 2 cases

The brain and acute leukemia cytoplasmic (BAALC; UniProt entry Q8WXS3) is a human protein of 180 amino acids. Eight alternatively spliced transcripts of *BAALC* were detected and five of them are described to form stable isoforms (isoform 1–3, 5 and 6) (Tanner et al. 2001). The remaining three splice variants encode the same predicted 80-amino-acid protein (isoform 4). These isoforms are expressed in cells of hematopoietic or neuroectodermal origin. The respective gene is located on chromosome 8q22.3 and is highly conserved in mammals and rodents (Fig. 1) but absent in lower organisms (e.g. Caenorhabditis, Drosophila). Currently, the function of BAALC is not fully characterized, but studies indicate a high clinical significance in pathological processes from several leukemias [Acute Lymphoblastic Leukemia (Kuhnl et al. 2010) and Acute Myeloid Leukemia (Baldus et al. 2003b; Bienz et al. 2005; Marcucci et al. 2005) to trisomy 8/Warkany syndrome 2 (Hemsing et al. 2019)].

In bone marrow, the *BAALC* gene was shown to be expressed mainly in hematopoietic progenitor cells and to be down-regulated during their differentiation (Baldus et al. 2003a). Its over-expression is strongly correlated in cytogenetically normal acute myeloid leukemia (CN-AML) (Weber et al. 2014; Zhou et al. 2015), that is more prevalent with progressing age, and associates with poor outcome questioning the correlation as a pure coincidence. Leukemia, in general, is a very heterogeneous hematological disease due to clonal proliferation of various undifferentiated progenitor cells. Therefore, understanding the signalling leading to myeloid (and/or lymphoid) progenitor cell proliferation and differentiation is indispensable to obtain a deeper understanding of leukemogenesis. Interestingly, *BAALC* over-expression also positively correlates with the *MN1* expression level (Heuser et al. 2012) but there is likely a common upstream regulatory mechanism. It was shown that BAALC does not enhance self-renewal of hematopoietic progenitor cells, but inhibits differentiation by desensitizing these cells to all-trans retinoic acid-induced proliferation arrest and differentiation, although, less effectively than MN1 does. Interestingly, *CEBPA*, one of the retinoic acid receptor target genes, is also a target of RUNX1 (alternatively AML1) (Friedman 2015). RUNX1 is a transcription factor important for hematopoietic cell development during embryogenesis (Tober et al. 2016) and as a hybrid protein formed by fusions of AML1 and ETO, a genetic aberration leading to the acute myeloid leukemia subtype M2 (Lin et al. 2017). In addition, RUNX1 can markedly increase the *BAALC* expression level if a certain SNP is located in the *BAALC* regulatory region (Eisfeld et al. 2014). The guanine-thymine exchange in this allele creates a binding site for the activating RUNX1 and predisposes the carrier to enhanced myeloid leukemogenesis. Thus, a high BAALC level is a risk factor for leukemogenesis. Whether the high *BAALC* expression is reason or consequence of this CN-AML subtype, can be clarified only by further investigations of the underlying molecular mechanism.

In rat, BAALC is membrane-anchored at its N-terminus via myristoylation at Gly2 and palmytoylation at Cys3 (Wang et al. 2005) and, due to its identical sequence, presumably also in human. Whereas the *BAALC* gene is studied regarding myeloid leukemia, its gene product, the BAALC protein, was neither characterized by biophysical nor biochemical methodology. This study presents ^1^H, ^15^N and ^13^C backbone resonance assignments to provide the basis for an atom-based structural view on the BAALC protein and its interactions employing high-resolution NMR spectroscopy.

## Methods and experiments

### Protein expression and purification

The full human *E. coli* codon optimized *BAALC* (isoform 1) gene (Tanner et al. 2001) was cloned into a pET28a plasmid using NdeI and XhoI restriction enzymes. The plasmid was modified using gene-tailor mutagenesis PCR using Platinum Taq DNA Polymerase (Invitrogen) to replace the thrombin by a TEV enzyme cut site between the His_6_-tag and the target gene. Therefore, the final protein contains an extra Gly residue at the N-terminus leading to the 181-residue-long protein. The 25 μl final reaction mixture contained 1 × High Fidelity buffer, 1 mM MgSO_4_, 0.2 mM dNTP, 0.6–0.6 mM forward and backward primers ~ 6.5 ng template (*BAALC* in pET28a between NdeI/XhoI) and 0.5 units of DNA polymerase. The following primers are used:

- fwd 5′ AGC AGC GGC CTG GTG CCG CGC GAA AAC CTG TAT TTT CAG GGC ATG 3′
- rev 3′ GTA GTG GTA GTG TCG TCG CCG GAC CAC GGC GCG 5′

After the initial 2-min-long denaturation, the reaction had 20 repetitions such as denaturation at 94 °C for 30 s, annealing at 65 °C for 30 s and extension at 68 °C for 6 min before the final round of extension at 68 °C for 10 min. The 10 μl purified DNA (PCRapate kit) was treated with 20 units DpnI in 1 × CutSmart buffer at 37 °C for 1 h to remove methylated DNA. The purified DNA (PCRapate kit) was transformed (~ 125 ng) into 50 μl DH5α *E. coli* competent cells using heat shock (10 min on ice and 45 s at 42 °C) and left on a LB kanamycin plate O/N at 37 °C. 147 ng/μl plasmid was purified from colonies and its insert was confirmed by DNA sequencing (Eurofins Genomics).

40–50 ng plasmid was used for heat shock transformation into 25 μl BL21(DE3) cells. Colonies were grown in 500 ml LB medium at 37 °C until OD_600_ reached 1.5. Cells were pelleted at 4800 rpm for 15 min using a Sorvall H6000A swinging bucket rotor (i.e. ~ 6700×*g*). The pellet was resuspended in 1 l sterile M9 medium supplemented with 1 g ^15^NH_4_Cl and 2 g ^13^C-labeled glucose. After one hour incubation at 18 °C, cells were induced by 0.3 mM IPTG O/N at 18 °C. Cells were lysed in 12 ml ice-cold lysis buffer (5 mM imidazole, 50 mM Tris and 300 mM NaCl, pH ~ 7.5, supplemented with proteinase inhibitor cocktail, DNAse I and 500 times diluted β-mercaptoethanol) three times using French Press. Cell debris was pelleted at 7600 rpm (Beckman Coulter C0650 rotor, i.e*.* ~ 5950×*g*) for 1 h. Supernatant was purified using Ni–NTA affinity chromatography. His-tagged BAALC was eluted using buffer containing 250 mM imidazole, 50 mM Tris, 300 mM NaCl and 1:500 β-mercaptoethanol, pH ~ 7.5 and its concentration measured by NanoDrop (Thermo Scientific). His-tag removal was conducted by using at least 40 × weight excess of 3 mg/ml TEV (200 μl) for about an hour at room temperature (~ 21 °C) and subsequently the sample was two times dialyzed using a 10 kD cut-off membrane against 1 l, 20 mM Tris, 5 mM NaCl, 2 mM DTT, pH ~ 7.5 at ~ 4 °C for about 2 h each. Due to low ionic strength precipitation of TEV occurred that was pelleted at 7600 rpm (Beckman Coulter C0650 rotor, i.e*.* ~ 5950×*g*) for 30 min. The clear supernatant containing the 181-residue-long BAALC (pI = 5.48) was further purified on anion-exchange chromatography using DEAE Sepharose resin (GE Healthcare) with gradual increase of NaCl concentration (20 mM, 30 mM, 40 mM and 50 mM) before the final 500 mM NaCl elution. Eluted fractions (at 50 mM NaCl) are mixed and dialysed O/N at ~ 4 °C against 20 mM Tris, 100 mM NaCl and 2 mM DTT, pH ~ 7.3. Using a 3 kD cut-off membrane, all dialysed protein was concentrated (7600 rpm Beckman Coulter C0650 rotor) until less than 1 ml.

The protein concentration was ~ 1.55 mg/ml measured by NanoDrop that was further purified with size-exclusion chromatography on S75 10/300 GL Superdex column (GE Healthcare) using an ÄKTA Avant system. Three 0.5 ml fractions containing significantly purified BAALC were concentrated again (7600 rpm Beckman Coulter C0650 rotor) and a 3 kD cut-off cassette dialysed O/N at ~ 4 °C against 20 mM sodium phosphate, pH 6.5.

The shorter BAALC isoform 6 was cloned from the isoform 1 construct by inserting a stop codon after position 54 and replacing Val54 by a glycine. Expression and purification was done as described. Deviating from the procedure described above, after His-tag removal by using TEV an additional Ni–NTA affinity chromatography was applied and the flow through was collected and concentrated.

### NMR spectroscopy

The NMR experiments for the ^1^H, ^15^N and ^13^C chemical shift assignments were acquired at 283 K in 20 mM sodium phosphate, pH 6.5 (90% H_2_O/10% D_2_O) on Bruker 600 (14.1 T) equipped with cryo-probe and 700 MHz (16.4 T) Avance III spectrometers. For sequential walk and backbone chemical shifts assignment, HNCO, HNCA (Kay et al. 1990), HN(CA)CO (Clubb et al. 1992), HN(CO)CA (Bax and Ikura 1991) and HNCACB (Wittekind and Mueller 1993) as well as (H)N(COCA)NNH (Bracken et al. 1997) experiments were employed. Additionally, assignments of side chain resonances were obtained from [^1^H,^15^N]-HSQC and [^1^H,^1^H,^15^N]-HSQC-TOCSY (Marion et al. 1989) and H(CCCO)NH and (H)C(CCO)NH (Montelione et al. 1992) spectra. The data was processed using TOPSPIN v.4.0.6 and analysed with CARA (Keller 2004) as well as CCPNmr Analysis (Vranken et al. 2005).

### Structure prediction

The secondary structure elements of BAALC were examined by analysis of the chemical shift data with the program CSI v.3.0 (Hafsa et al. 2015) and the secondary structure propensity approach (Marsh et al. 2006). For the sequence-based prediction the IUPred2A server was used (Dosztányi 2017; Mészáros et al. 2018).

## Extent of assignments and data deposition

In contrast to the wild type, the BAALC protein used here for the NMR experiments exhibits one additional N-terminal amino acid (Gly0) arising from cloning purposes. This Gly0 is not considered in the following statistics.

**Fig. 2:**
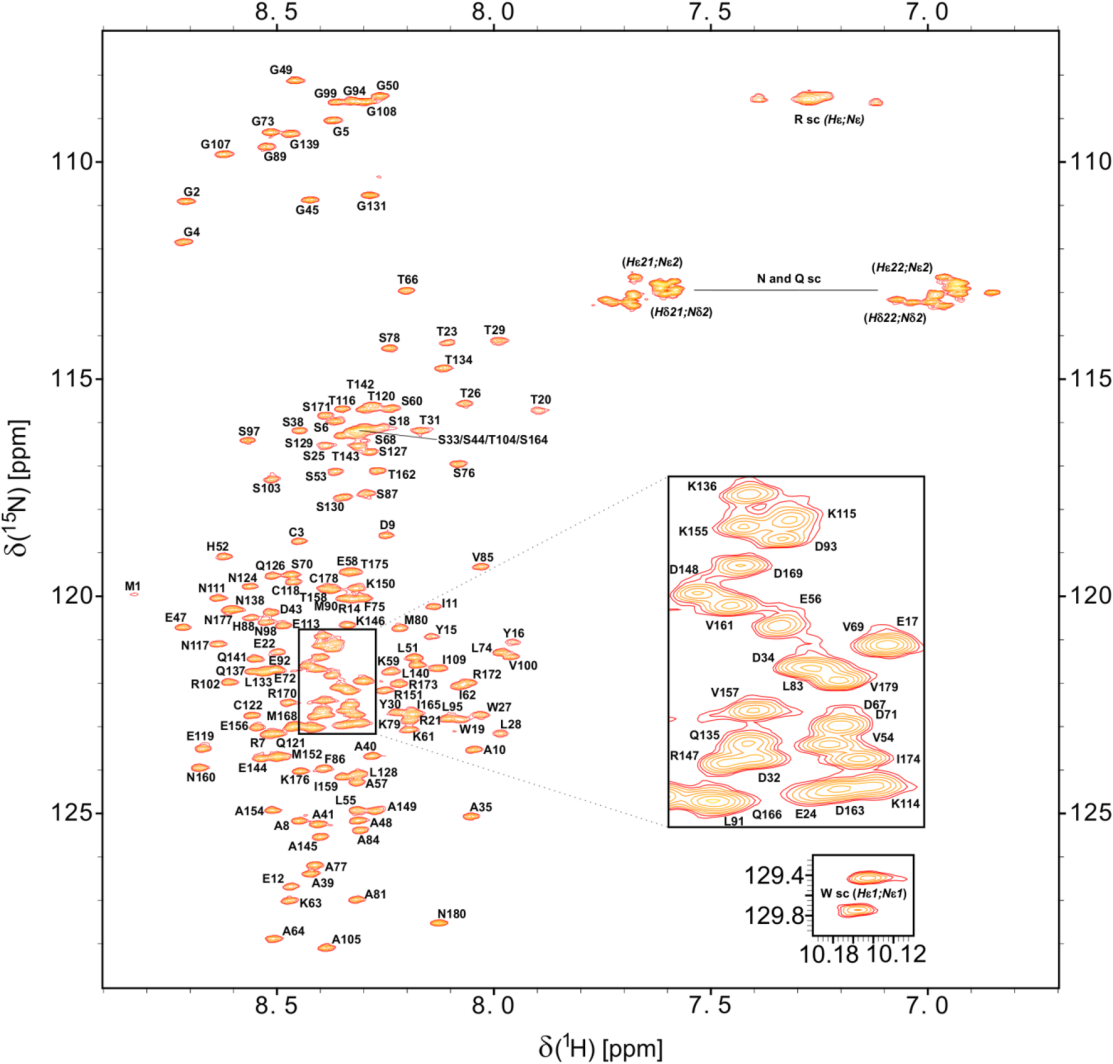
[^1^H, ^15^N]-HSQC spectrum of ^15^N-labelled BAALC at pH 6.5, 283 K. Assignments for backbone amides are annotated. Non-degenerate protons of the side chain amino groups are connected by a shaded line. Figure prepared using Sparky (T. D. Goddard and D. G. Kneller, SPARKY 3, University of California, San Francisco)

The [^1^H,^15^N]-HSQC spectrum of BAALC (Fig. 2) allowed assignment of 99% of the backbone ^1^H^N^ (163/164) and ^15^N^H^ (163/164) resonances of the non-proline residues. Only the resonances of Gln167 of BAALC could not be determined. 100% of the ^13^C^α^ (180/180) and 99% of the ^13^C′ (except Pro36) backbone chemical shifts were assigned employing HNCO, HN(CA)CO, HNCA, HN(CO)CA and HNCACB spectra. 99% of assignments (164/166) for the β-carbon resonances were achieved (except: Met1, Arg21). In addition, we report 14 ^13^C^γ^ resonances out of the 16 proline residues which allow to predict all the respective prolines possessing a trans conformation (^13^C^γ^ ~ 27 ppm with ^13^C^β^ ~ 32 ppm shifts) (Schubert et al. 2002). Furthermore, 98% (191/194) of the H^α^ resonances were assigned. Only the H^α^ nuclei of Arg14, Gln167, Met168 could not be assigned.

**Fig. 3:**
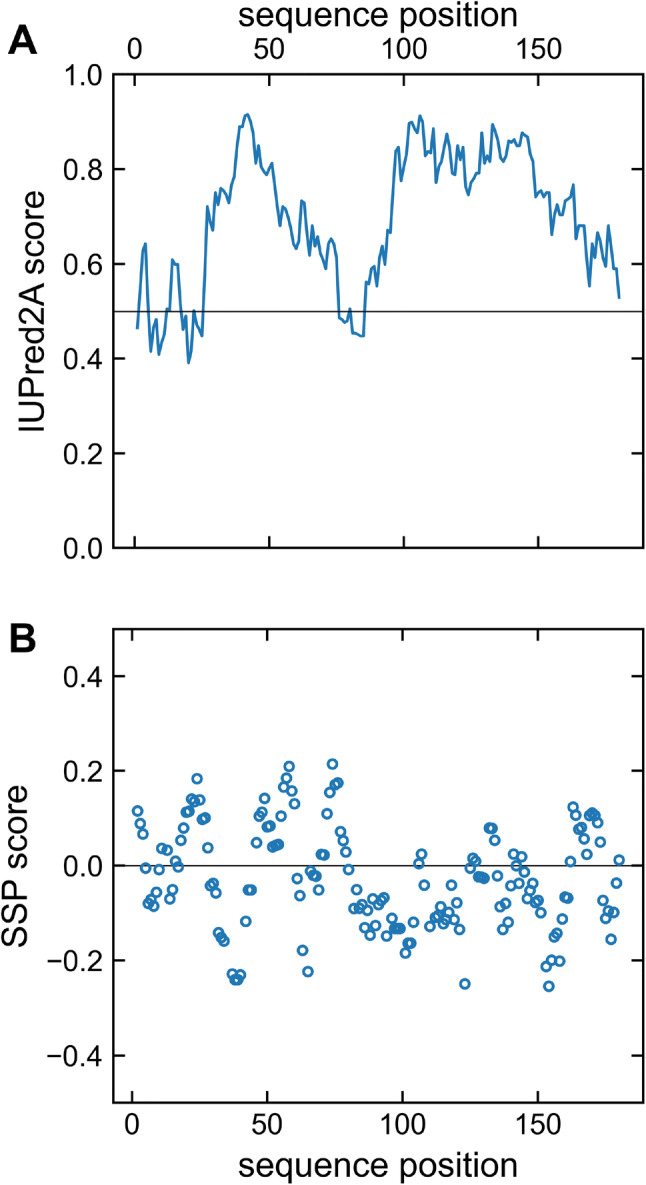
**A** IUPred2A prediction of BAALC indicating the disordered nature of this protein. The residue-specific IUPred2A score for BAALC is indicated as solid line. Values higher than the cut-off (0.5) indicate disordered segments, lower values predict structured regions. **B** The sequence specific secondary structure propensity (SSP) scores are presented (open circles). Values below 0 represent β-structure propensity. Helical propensity is indicated by positive values. At a given residue a SSP score of 1 or − 1 reflects fully formed α- or β-structure, respectively. The SSP script was used with the default setup and, as recommended for disordered proteins, only C^α^, C^β^ and H^α^ chemical shift were applied

Analysis of structural elements by the CSI web server (data not shown) resulted in an all-coil prediction. This supports the expectation based upon the appearance of the [^1^H, ^15^N]-HSQC spectrum (Fig. 2) which showed a reduced spectral dispersion of average chemical shifts implying flexibility typical for intrinsically disordered proteins. An IUPred2A analysis (Dosztányi 2017; Mészáros et al. 2018) also predicts that BAALC is predominantly disordered with very weak, short ordering tendency at residues 6–11, 18–25 and 76–85 (Fig. 3A). In order to reveal potential secondary structure propensity (SSP), which might not be detected by the other approaches, we analyzed the chemical shift data using the SSP method (Fig. 3B) (Marsh et al. 2006). By averaging the potential α-helical and β-sheet regions of the calculated SSP scores, an overall total of 6.3% α-structure and 3.3% β-structure, respectively, is estimated for BAALC. The large degree of disorder/flexibility is consistent with the findings of the other structure prediction tools and confirms the observation made from the ^1^H^N^, ^15^N^H^ chemical shift dispersion.

**Fig. 4:**
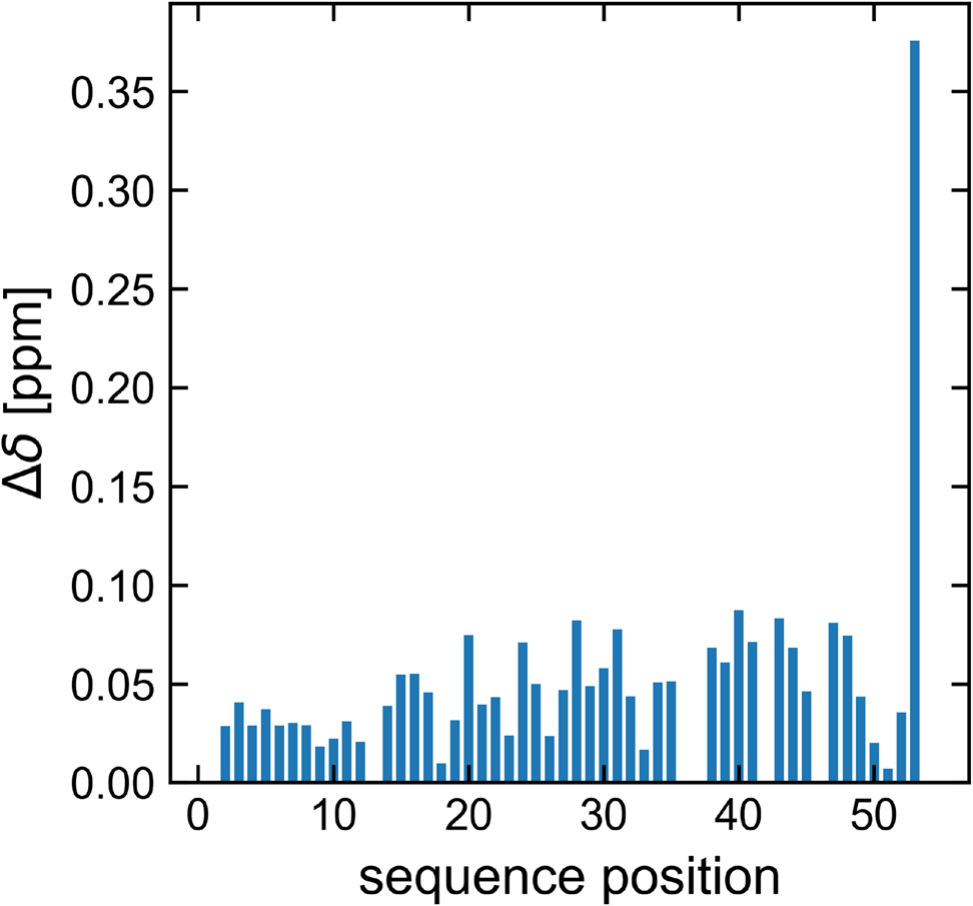
Combined ^1^H^N^ and ^15^N^H^ chemical shift comparison of BAALC isoform 1 and 6 based on [^1^H, ^15^N]-HSQC spectra recorded at pH 6.5, 283 K. Resonance shift changes are minor (less than 0.1 ppm) except that for residue 53, which is the penultimate residue of isoform 6. Note, residue 54 having the largest change is not shown as these represent different types. Combined chemical shift is given by Δδ = [(Δδ^2^_HN_ + (Δδ_N_/6.5)^2^)]^½^ according to (Mulder et al. 1999)

A ^1^H^N^ and ^15^N^H^ chemical shift comparison of BAALC (isoform 1) with its shortest, neuroectodermal isoform 6 was performed (Fig. 4). The result indicates that only minor changes (less than 0.1 ppm) in the ^1^H^N^ and ^15^N^H^ chemical shifts occur. The only exception is residue 53, which can be explained by its penultimate position in isoform 6.

As described above, it is likely that also the human BAALC protein is anchored in the membrane. The same applies to post-translational modifications (e.g. phosphorylation at some Ser, Thr and Tyr residues). It remains to be investigated whether the spatial proximity of the BAALC protein and its shorter isoforms to a membrane and/or additional modifications causes structural changes.

The ^1^H, ^13^C and ^15^N backbone chemical shifts of BAALC have been deposited in the BioMagResBank (BMRB) under the accession number 28084.
